# Confection Confusion: Interplay Between Diet, Taste, and Nutrition

**DOI:** 10.1016/j.tem.2020.11.011

**Published:** 2020-12-28

**Authors:** Christina E. May, Monica Dus

**Affiliations:** 1 Neuroscience Graduate Program, The University of Michigan, Ann Arbor, MI, USA; 2 Department of Molecular, Cellular and Developmental Biology, The University of Michigan, Ann Arbor, MI 48109, USA; 3 Current address: Neuroscience Institute, the NYU School of Medicine, New York, NY 10016, USA; 4 https://sites.lsa.umich.edu/dus-lab/

## Abstract

Although genetics shapes our sense of taste to prefer some foods over others, taste sensation is plastic and changes with age, disease state, and nutrition. We have known for decades that diet composition can influence the way we perceive foods, but many questions remain unanswered, particularly regarding the effects of chemosensory plasticity on feeding behavior. Here, we review recent evidence on the effects of high-nutrient diets, especially high dietary sugar, on sweet taste in vinegar flies, rodents, and humans, and discuss open questions about molecular and neural mechanisms and research priorities. We also consider ways in which diet-dependent chemosensory plasticity may influence food intake and play a role in the etiology of obesity and metabolic disease. Understanding the interplay between nutrition, taste sensation, and feeding will help us define the role of the food environment in mediating chronic disease and design better public health strategies to combat it.

## Introduction

‘…having bowed to the inevitability of the dictum that we must eat to live, we should ignore it and live to eat…’ – M.F.K. Fisher, *An Alphabet for Gourmets*

The chemosensory system is the key to unlock many of life’s daily pleasures: the complexity of chocolate, the nuttiness of aged cheese, or the sweetness of fruit. In particular, the sense of taste is critical to the detection of palatable qualities in food, which is why it plays such an important role in food intake and body weight. The preference for some taste qualities over others, such as sweetness over bitterness, is largely genetically encoded, likely the result of our ancestors’ adaptive association of taste cues with good experiences such as nutrient density, or bad ones, like malady and death [1]. However, many other taste preferences, such as our liking for coffee or vegetables, are not innate, and are instead learnt through experience [2]. Thus, the taste sensations of animals exist in this narrow space between nature and nurture, shaping their likes, loves, and dislikes. Overlaid on this, is the fact that exposure to different foods can also reshape taste sensation. Studies in humans, rodents, and insects have found that the **sensitivity** (see Glossary) and **intensity** of taste sensation changes with **diet composition** [1]. For example, insects feeding on bitter food sources downregulate the perception of bitterness [3,4] and humans eating low-sodium diets over time show increased preference for lower concentrations of sodium [5]. Thus, the chemosensory system is plastic and able to tune its receptive properties to the dietary environment. This plasticity of the taste system – a property shared by many sensory apparatuses – allows animals to detect and select food sources in a range of ever changing dietary environments that may be quite different from the original ecological niche in which the sensory system developed and evolved.

Given the importance of taste in feeding behavior, a question that immediately arises is how this diet-dependent **chemosensory plasticity** influences food preference, choice, and overall energy intake. Indeed, changes in the **sensation** and **perception** of the orosensory properties of food could profoundly alter eating habits in ways that influence weight gain and the risk of metabolic disease. This question is particularly relevant in the current food environment, since manufactured and processed foods contain high amounts of salt, sugar, and fat that appeal to our preferences and also reshape the way we taste (and likely the way we feel) [6–8]. What is then the interplay between diet exposure, chemosensory plasticity, and eating behavior? Here, we review the evidence for how diet exposure alters taste sensation and perception and discuss the potential molecular and neural mechanisms through which this occurs. Since the effects of dietary fat and salt on taste have been already reviewed [9,10], we primarily focus on the role of dietary sugar in sweetness perception. We report the findings from recent studies in humans, rodents, and invertebrates, discuss open questions and future directions, and propose ways in which diet-induced chemosensory plasticity could affect feeding behavior and impact the risk for metabolic disease.

## Added Sugar

Sugar is a naturally occurring component of many foods, such as fruit, that increases their nutrient properties and provides pleasant sensory qualities. However, sugar is also added to foods in the forms of syrup or powder during their processing and preparation. Because sugar has Food and Drug Administration (FDA) generally recognized as safe (GRAS) status, there is no limitation for sugar content in foods, other than current good manufacturing practices (https://www.accessdata.fda.gov/scripts/cdrh/cfdocs/cfcfr/CFRSearch.cfm?fr=184.1854). The lack of recognized standards for sugar content in food, together with the fact that flavor is the primary driver of eating choices in consumer surveys (https://foodinsight.org/2020-food-and-health-survey), has led to high levels of added sugar in food. In the USA, ~80% of grocery store foods contain added sugar [11] and food deserts in rural and urban areas make access to unprocessed food challenging [12,13]. Worldwide, added sugar consumption is higher than that recommended both among adults and children [14,15], and even countries with food insecurity face the double burden of malnutrition, as many of the foods available are processed, nutrient poor, and high in added sugar and fat [16,17]. Added sugar is associated with higher caloric intake [14,18–20,97], weight gain, obesity [21,22], and a whole host of metabolic-related diseases, from diabetes and heart diseases to cancer and neurodegeneration [14,23]. Given its prevalence in our food supply and its concerning effects on our bodies, understanding how high levels of sugar in our diets affect taste sensation and feeding behavior is paramount to design public health interventions to curb the spread of metabolic disease.

## Diet and Chemosensory Plasticity

Studies from humans to insects have shown that diet can shape flavor preferences and potentially skew food choice. The best understood example of diet-induced taste plasticity in humans is that of salt, where a gradual reduction in the sodium content of the diet led to higher perceived intensity and preference for lower salt concentrations [5,24,25], while an increase in dietary salt intake shifted the concentrations for maximum pleasantness upward [26]. Similarly, consumption of a high-fat diet has been linked to a decrease in fat sensation and higher preference for fatty foods [10,27,98]. Thus, it is reasonable to hypothesize that the levels of dietary sugar may similarly influence sweet taste.

There are a few challenges with tackling this question in the context of human daily diets. First, it is hard to accurately quantify total amounts of dietary sugar in human diets. Sugar is a naturally occurring component of foods, but also a food product (added sugar) that is listed under dozens of different names and does not have a percent daily recommended dose in food labels [28,29] (although recently food labels carry the ‘added sugar’ line). Second, sweetness in food comes not only from sugar, but also from noncaloric sweeteners, so the sources of sweetness have both caloric and noncaloric value, which complicates the analysis. Third, studies that investigate the effects of high sugar exposure in the medium and long term in humans are potentially unethical, because high levels of caloric sweeteners contribute to severe metabolic consequences. Finally, the anatomy of the mammalian taste system is complex, and human studies are limited to measuring taste sensation and preference using psychophysical and hedonic scales (Boxes 1 and 2), which reveal little about the underlying neural and molecular mechanisms at play. Together, these points make assessing the overall impact of high sugar diet in humans difficult. Some of these challenges can be tackled in laboratory-based studies, where sugar types and levels can be controlled by researchers. Others, such as the questions of exposure and mechanisms, are better addressed with the use of different animal models which offer different approaches and advantages (Boxes 1 and 2). We review human and animal studies (Table 1), showing how addressing experimental points across organisms leads to an integrated understanding of diet and chemosensory plasticity.

Most laboratory studies in humans have studied the impact of sugar levels on taste by replacing it with other macronutrients, or decreasing overall calorie intake in the long or short term. In a hallmark study using a randomized clinical trial, Wise and colleagues [30] placed human subjects on a reduced-sugar diet for 2 months, substituting calories from sugar with fat, proteins, and complex carbohydrates. The authors found that these individuals had higher intensity ratings for sweetness by the general Labeled Magnitude Scale (gLMS), uncovering a negative association between sugar levels and sweetness intensity. Other studies also support this idea, showing that sweet taste sensitivity increased when sugar levels were reduced by fasting and caloric restriction in lean [31] and obese adult subjects [32,33,99], but not in children with high BMI [34]. Thus, similar to salt, there appears to be a strong relationship between lower sugar levels in the diet and an increase in intensity and sensitivity for it.

Conversely, higher levels of dietary sugar may lead to a decrease in the perception of sweetness. Indeed, dietary intake studies indicate that higher consumption and frequency of sugar intake from baked goods, sweetened beverages, and convenience foods were associated with lower sweet taste intensity by gLMS [35,36] and sensitivity (recognition threshold) [37] (Box 2). A strong negative relationship between sweet taste intensity (measured by recalled ratings) and sweetened beverage consumption has also been described [38]. These findings are in line with a controlled dietary intervention study which found concentration-specific decreases in sweet taste intensity (by gLMS) and pleasantness when the subjects’ diet was supplemented with a sweetened soft drink for 1 month [39]. Research comparing sweetness taste thresholds in populations with different levels of sugar in the diets (i.e., rural vs urban) has also pointed to a generally inverse correlation between dietary sugar and sweet taste sensitivity [31–33]. Together, these studies have suggested that the sensation and preferred levels of sweetness may depend on a person’s diet.

Studies in animal models have helped substantiate this idea. First, as in human studies [30], consumption of a reduced-sugar diet led to an increase in sensitivity of the gustatory cells and higher **proboscis extension response** (PER; see Boxes 1 and 2 for anatomy and assays) to lower concentrations of sweet in *Drosophila melanogaster* flies [40]. Conversely, exposure to a high-sugar diet (10, 20, and 30% sucrose, glucose, or fructose) resulted in lower taste sensitivity and intensity (assayed by food detection tests and PER) after 3–7 days, and these changes in taste sensation were independent of weight gain [20,41]. A decrease in sweet taste responses was specific to caloric sugars and did not occur with dietary supplementation of the noncaloric sweetener sucralose [20]. These alterations in sweet taste were due to changes in the responsiveness of the sweet taste receptor neurons to sweet stimuli as measured via extracellular recordings of the sensilla stimulated with sucrose [20,40]. Moreover, *in vivo* imaging of the sweet taste neurons presynaptic terminals (which synapse in the brain) showed a decrease in calcium responses [20] and vesicular release [42] (for anatomy, see Box 1). Together, data from insects using behavioral assays of taste sensitivity and intensity, electrophysiological recordings, and *in vivo* imaging argues that dietary sugar content bidirectionally shapes the way animals taste sweet.

Although the dietary manipulations in rodent models are not limited to just increasing and decreasing sugars, they also support this idea. First, rats fed a high-sucrose liquid or dry diet (30% sucrose in addition to no-sucrose chow or a 66.6% kcal sucrose chow, respectively) for 40 days had lower **chorda tympani** (CTs; Boxes 1 and 2) responses to 1 M sucrose [43]. Consistent with this, rats fed a high-fat and high-sugar diet (45% fat and 17% sugar) for 8 weeks presented dulled **nucleus tractus solitarius** (NTS; Boxes 1 and 2) responses to taste that included lower magnitude and duration and longer latency to sweet solutions and naturalistic taste stimuli [44]. However, these changes seem to require an extended exposure to dietary sugar because previous work reported an increase in CT responses to sucrose and no changes in the NTS responses following a short, 3-day access to a high-energy diet (45% fat and 17% sugar) [45] or to a 0.5 M sucrose solution [46], respectively. These findings are especially interesting when compared with those of two studies in which mice fed a high-fat diet (60% fat and 22% carbohydrates) for 8 weeks showed a decrease in the number of taste buds responsive to sweet stimuli (saccharin and Acesulfame K) and in the amplitude of their calcium responses measured *ex vivo* with calcium-sensitive dyes [47,100]. As shown in genetic studies in *D. melanogaster* [20], some of these changes in responsiveness occurred independently of diet-induced obesity [100]. Importantly, in rodents and flies, these physiological alterations were accompanied with behavioral changes in licks, feeding behavior, and food preference [20,47,48,44,100].

Together, evidence from flies and rodents suggest that the levels of sugar in diet can directly reshape the gustatory system and affect the detection, transduction, and processing of sweetness, impacting taste sensation and food preference. However, the amount of sugar in the diet, the content of other synergistic tastants like fat, and the length of exposure seem to be critical factors in the reshaping of sweet sensation. This may help explain conflicting evidence from previous work on changes in taste function with obesity in humans, where high **body mass index** (BMI) has been associated with an increase in sensitivity and intensity [49–51], a decrease [39,52–58], or no changes [59,60]; while in these studies diet was not investigated, it may have played a role in the results.

## Mechanisms of Diet-Induced Chemosensory Plasticity

In the human salt studies, changes in salt concentration pleasantness and perceived intensity only occurred when subjects were given extra salt in their food, rather than supplemented with untasted salt tablets [26]. This line of evidence suggests that the taste system has to be engaged for the effects of diet to manifest. This question has not been addressed in animal studies, which have instead investigated changes in the expression of genes important for taste function [20,47,48,44,100] and changes in the anatomy of the taste system with diet. Several experiments found that the mRNA levels of α-gustducin and **phospholipase C-β2** (PLC-β2) were decreased in mice fed a high-fat diet [47,100,101]. This result is interesting when compared to human studies that measured lower expression of PLC-β2, **transient receptor potential M5** (TRPM5) and the **taste 1 receptor member 2** (TAS1R2) in the fungiform papillae of subjects with obesity [56], and an increase in DNA methylation at these genes from blood samples of people with high BMI [61]. However, the diet of humans was not monitored [56,61]). In terms of anatomical changes, three studies observed no changes in the number of taste buds and taste cell types in rats [43] fed sucrose or mice fed high-energy diets [47,100]; however, two studies from a separate group reported a decrease in the number of circumvallate and fungiform papillae and in the markers of taste receptor cell (TRC) proliferation in mice fed high-fat diets ([62], 2020), although the latter was attributed to inflammation due to obesity.

Research in *D. melanogaster* flies, where the genetic dissection of molecular mechanisms is more amenable, has uncovered several conserved avenues of investigation. Our group identified the hexosamine biosynthesis pathway – a signaling pathway involved in the pathophysiology of metabolic disease in mammals – as the molecular link between high sugar intake and the responsiveness of the sweet gustatory neurons [20]. Specifically, we found that high levels of sugar enhance the activity of the enzyme O-GlcNAc transferase, which resulted in lower responses of the taste cells to sweet. Recently, our group also mapped the transcriptional and chromatin changes that occur with high levels of dietary sugar in the sweet taste cells. We discovered that the conserved gene silencing **Polycomb repressive complex 2** mediates the effects of the dietary environment on taste function by decreasing the expression of developmental genes required to mature and wire the taste cells [41]. There were no changes in the number of sweet TRCs in flies [20]. Another group also identified transcription-based mechanisms based on the conserved **peroxisome proliferator-activated receptor γ coactivator 1α** (PGC1α) regulators for sugar sensitivity in flies deprived of sugar [40].

Converging evidence from human, rodent, and insect molecular and cellular studies hints that sugar and high-energy diets shape taste function via changes in gene expression initiated by diet, and by decreases in taste stem cell division mediated by inflammation due to obesity.

## Diet-Dependent Chemosensory Plasticity, Feeding Behavior, and Obesity

**Figure 1.**
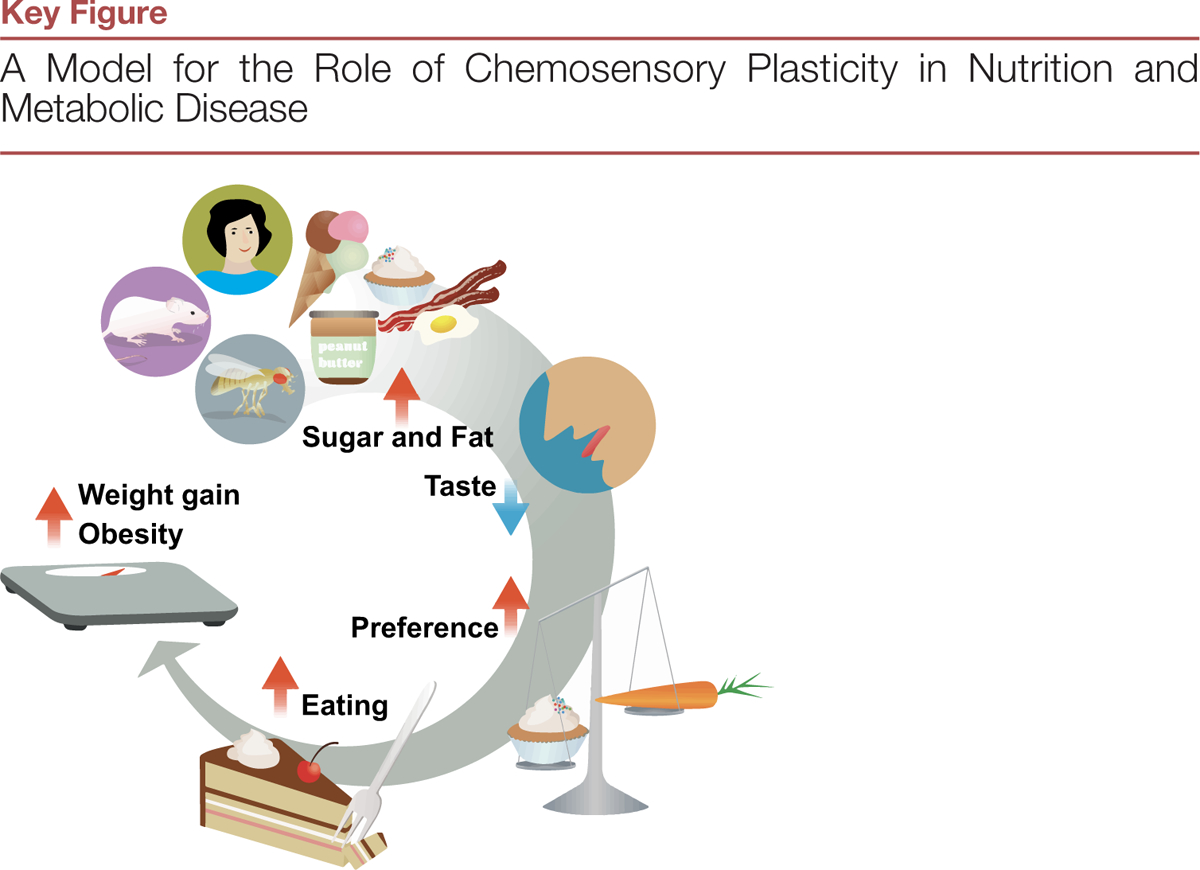
Consumption of high-sugar and high-fat foods reshapes taste sensation and preference in rodents, humans, and insects, although the strength of the evidence differs across these organisms. These changes could alter different aspects of food intake, such as compromising the nutritional evaluation of food, inhibiting sensory enhance satiation, or creating a reward deficit. Together, these alterations could bias selection towards some types of foods over others and promote food intake, creating a vicious, reinforcing cycle that, over time, leads to weight gain and increases the risk of metabolic disease.

Data from animal models and humans suggest that dietary exposure to high sugar, salt, or fat shifts preference to higher concentrations [5,20,26,27,40,41,44,47,63], which could bias organisms towards the selection of foods with higher levels of these compounds (Figure 1, Key Figure). Because these foods are more calorically dense, this could promote weight gain and metabolic disease, which may further disrupt chemosensory and reward circuits to reinforce their selection. Two studies in flies also showed that preventing the diet-dependent dulling of taste protected animals from overfeeding and weight gain [20,41], showing that chemosensory plasticity causes changes in feeding behavior in this model organism.

In addition to a shift in preference, changes in responsivity of the gustatory system could play a role in food intake and obesity by disrupting sensory associations. Sensory signals function as cues that predict their filling power [64–66] and slow down the meal episode before nutrient signals consolidate **satiety** [67], a phenomenon known as **sensory-enhanced satiety** [64]. Sensory intensity is positively correlated with food-satiating power [66,68]. One way in which a decrease in the intensity of taste sensation could lead to higher feeding is an impairment in sensory satiety, which would occur if the prediction about the sensory cue is not updated. In this case, the sensory cue (sweetness) would predict a lower caloric density than it used to, prompting the animal to extend the meal episode to match its expected energy needs. Variations from expected taste signals activate reward (ventral striatum) and flavor processing areas (anterior dorsal insula) in the human brain [69], which typically results in updated predictions about the sensory cue value. However, reinforcement learning, and particularly the negative outcome learning that could occur when taste sensation changes, is impaired in humans with obesity [70] and rats fed a cafeteria diet [71], and these rodents show attenuated sensory satiety [42,71]. Daily consumption of ice cream is associated with lower activity of the striatum and insula to milkshake receipts in humans [72]: the dulling of the chemosensory system to sweet and/or fatty stimuli could contribute to this phenotype. To this point, a recent study from our laboratory found that in *D. melanogaster* deficits in the transmission of the taste signal out of the TRCs led to decreased and delayed processing of sweetness by dopaminergic neurons, which impaired with satiety and caused higher food intake [42].

Finally, diet-dependent chemosensory plasticity could also influence food intake by falsely amplifying the energy reward from the food. Studies in humans [73], rodents [74], and flies [75] have shown that a mismatch between sensory cues about energy and the energy acquired from food alters the reinforcing properties of the food and promotes intake. A mismatch between a sensory cue, diminished by diet, and the predicted energy content, would result in the animal receiving a greater nutrient reward than expected. This higher expectation would increase the strength of the sensory cue in future meals (**incentive sensitization**) and sensitize animals to sugar, prompting increased food intake. This hypothesis is in line with a vast number of experimental results that show higher responses to cue presentation with high energy diet and obesity [70,76,77].

## Concluding Remarks and Future Perspectives

Converging evidence suggests an inverse relationship between sugar levels in diets and sweet taste sensation. In particular, data from animal models argue that sugar, fat, and high-energy diets remodel the chemosensory system at the level of the taste receptor cells, the sensory neurons, and the circuits processing taste in the brainstem and the central brain. In addition to their direct effects on the sensory system, high-energy diets (sugar, fat, or a combination of both) can also exacerbate taste dysfunction by promoting weight gain, obesity, and inflammation. While the mechanisms are still unclear, studies suggest this occurs via changes in gene expression initiated by diet, and by decreases in taste stem cell division mediated by inflammation due to the obese state (Outstanding Questions). Of note, our recent discovery that in flies sugar metabolites epigenetically reprogram the expression of neurodevelopmental pathways responsible for the wiring of the sensory cells and nerves provides an important starting point to study effects of diet on the mammalian taste system. Finally, apart from work in invertebrates in which a dulling of sweet taste causes lower satiation, higher food intake, and weight gain [20,41,42], we still know little about how diet-dependent chemosensory plasticity influences food preference, choice, and intake in mammals. Studies that use neuro-, chemo-, and optogenetic tools, similar to those used in *D. melanogaster*, will allow researchers to directly assess the contribution of taste plasticity to feeding behavior and experimentally test the model proposed in Figure 1.

In conclusion, sugar content, and high-energy diets more broadly, can alter taste sensation at the molecular, neural, and behavioral levels, both directly and indirectly. However, the majority of these findings comes from animal models, and thus clinical studies that measure sweet taste plasticity in response to controlled dietary sugar levels in humans are essential. In particular, given the role taste plays in feeding behavior, research on the interplay between nutrition and sensory neuroscience will be a key foundation to design public health strategies [78] to reduce the levels of salt, sugar, and fats in our diets and curb the spread of metabolic disease.

## Figures and Tables

**Figure I.**
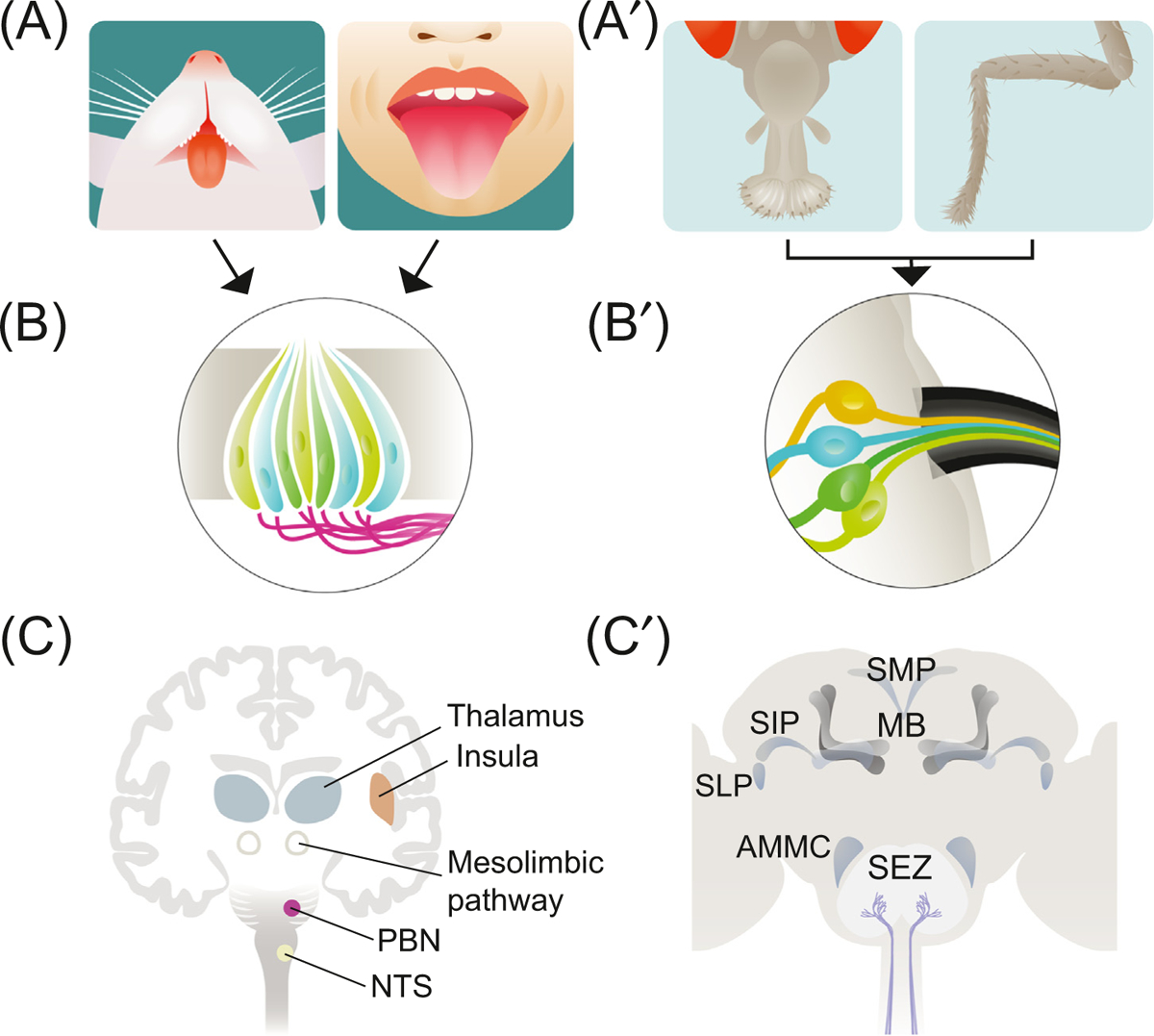
Comparative Anatomy of Taste in Mammals and Insects. (A, A′) Dedicated organs for taste sensation are located on the tongues of mammals (A, rodents, left, and humans, right) and on the proboscis and other body parts, such as the legs, of insects (A′). (B, B′) Specialized cells express taste receptors in the taste organs. (B) In the mammalian tongue, three types of taste cell (light green, dark green, and blue) are organized into clusters called taste buds and contact nerve fibers projecting to the brain (magenta). In the insect, taste hair (sensilla) house three or four single-modality taste neurons (light green, dark green, blue, and yellow) that project to the SEZ. (C, C′) Brain structures for taste sensation, processing, and reward in mammals and insects, here shown is the *Drosophila melanogaster* brain. Abbreviations: AMMC, antennal mechanosensory and motor center; MB, mushroom body; NTS, nucleus of the solitary tract; PBN, parabrachial nucleus of the pons; SEZ, subesophageal zone; SLP/SIP/SMP, superior lateral/intermediate/medial protocerebrum.

**Table 1.** Summary of Findings on the Effects of Obesity and Diet on Sweet Taste and Processing

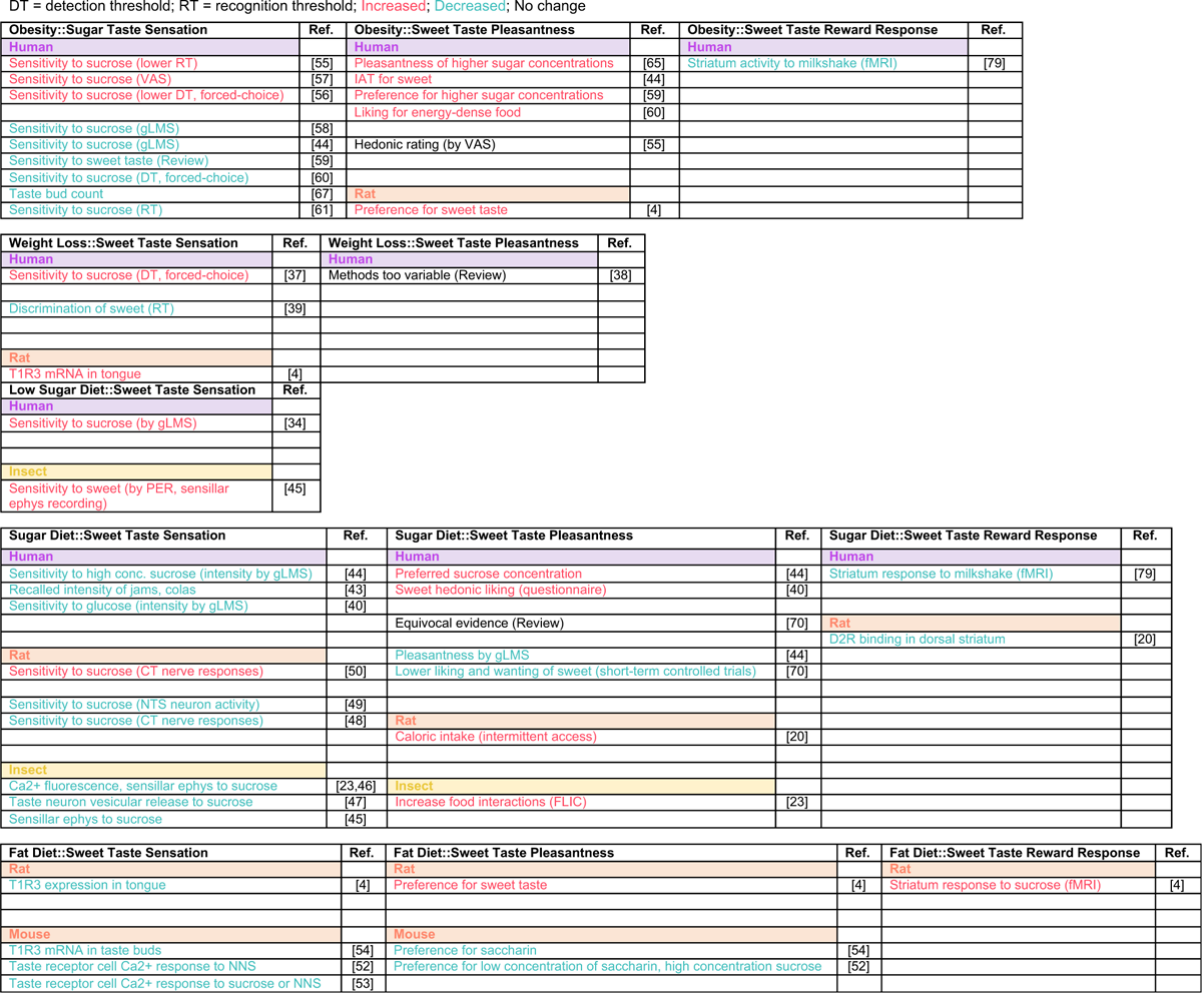

## Highlights

Taste sensation varies with age, disease state, and environmental exposure. Emerging data from animal models suggest that diet composition, particularly the levels of sugar and fat, alter sweet taste sensation to influence feeding behavior and the risk of metabolic disease.

Diet composition and obesity contribute to different aspects of chemosensory plasticity.

Consumption of high sugar and/or high-fat diets reshapes the transcriptome and epigenome of the taste cells and nerves and dulls their responsiveness to stimuli.

Sugar levels in the diet also affect taste sensation in humans, but more research is needed to understand the impact of high sugar intake on taste and feeding.

## Outstanding Questions

How do sugary and high-energy diets change the expression of genes necessary for sweet sensation in TRCs? One possibility is that the levels of sugar and its byproducts are directly coupled to transcriptional mechanisms in the TRCs, since metabolites can influence chromatin state and gene expression and the levels of sugar modulate pathways that post-translationally modify histones and other cellular proteins with acetylation, methylation, and GlcNAcylation or directly methylate DNA or RNA. A second possibility is that the metabolic side effects of sugar and high-energy diets, such as endocrine dysregulation and inflammation, could also impact gene expression, signaling pathways, and stem cell renewal, and hormonal signals, which are known to influence taste function.

Are disruptions in taste temporary, or do they persist upon shifts in the dietary environment, as work from insects indicates? The recent discovery that dietary metabolites engage neurodevelopmental programs to rewrite and rewire the adult taste system provides a mechanism to induce lasting effects in the taste cells or sensory nerves.

What is the impact of alterations in the TRCs or the sensory nerves on the neural processing of taste, flavor, and reward in the brain? We do not know if the changes in the NTS of rats on high-energy diets are due to plasticity in the TRCs and the CT nerve, or the result of cell-autonomous effects of diet on this brain region. Other brain circuits, such those involved in reward, could also be affected by enhancements/deficits in sweetness processing. Specifically, distortion in the responsiveness and activation of dopaminergic circuits with diet and high BMI have been extensively documented in mammals. It is plausible that at least some of these alterations could occur because of diet-driven impairments in gustatory processing. Studies that tackle the network effects of chemosensory plasticity will help answer these questions.

Does plasticity in one taste modality influence taste and flavor perception more broadly? Since processed food is nutrient poor but has high content of salt, sugar, and fat, changes in the detection and processing of each of these modalities could have synergistic effects and impact the sensory perception of healthy foods that may have bitter qualities, like vegetables. Thus, understanding how distortions in one modality may alter to the perception of others will be an important aspect of future studies.

## Box 1. Anatomy of Sweetness

Due to convergent evolution, the anatomy of sweetness is remarkably similar between insects and mammals [79,80]. Taste organs in or around the oral cavity express chemoreceptors for the detection of sweetness and other qualities (Figure IA-A′) [79,80]. In the mammalian tongue, taste buds contain different types of TRCs: type II taste cells detect sugars due to the [81] expression of the G-protein-coupled TAS1R2 and TAS1R3 subunits (Figure IB). Binding of sugars to these receptors activates the second messenger PLC-β2, which triggers calcium release from intracellular stores [81] and opens TRPM4 and TRPM5, leading to membrane depolarization and release of ATP through the CALHM1/CALHM3 channel onto the local nerve that innervates the taste bud [81–83]. In lieu of taste buds, insects have taste sensilla (hairs) outside and inside the proboscis as well as in other body parts, such as the legs [79,80]. Each sensillum contains chemosensory nerve endings for four taste neurons, including one sweet-sensitive neuron [79] (Figure IB′). In *D. melanogaster* each sweet taste neuron expresses a subset of the adult fly’s eight sweet taste receptors [79], which are thought to be ligand-gated ion channels based on the structure of a related olfactory receptor [84]; yet, G proteins are also required for the sensing of sweet [85].

From the oral cavity, sensory information travels into the brain [79,80] (Figure IC-C′). In mammals, the CT and glossopharyngeal nerves (cranial nerve IX) carry taste information to the NTS in the brainstem, with further relays into the parabrachial nuclei, the gustatory thalamus, and the primary gustatory cortex in the insula. Sweet taste signals also activate reward circuits in the ventral tegmental area [80] and are associated with dopamine release in the ventral striatum [86]. In insects, sweet taste neurons project to the **subesophageal zone** [79], and are then relayed via different neural tracks to parts of the brain involved in multisensory integration, reward, and energy balance. Like in mammals, the sweet signal activates a distinct subset of dopaminergic neurons [87,88].

## Box 2. Assaying Taste

### Humans

Sensitivity assays test detection and recognition threshold by giving subjects different concentrations of tastants. Intensity assays used semantic scales such as the Visual Analog Scale, the Labeled Magnitude Scale, and the gLMS to determine how strongly the subject perceives the stimulus. Hedonic measurements assess a person’s liking of a stimulus and their preference for different concentrations and are done with psychophysical assays, tests, or scales, including the Implicit Association Test (IAT) questionnaire and the Palatable Eating Motives Scale (PEMS). Food-intake measures occur inside and outside the laboratory; in the latter they use food frequency questionnaires or dietary recalls, although these may not accurately reflect diet composition or food intake [89]. Finally, imaging techniques like functional magnetic resonance imaging and positron emission tomography measure the activity of brain areas involved in taste processing and reward (e.g., NTS, insula, and striatum).

### Animal Models

In rodents taste sensation is estimated with brief-access lick tests, which measure the number of licks an animal makes for short 5–30-s trials after a taste stimulus is presented [90], while in insects, the PER measures the magnitude of proboscis extension when the taste cells are stimulated with a tastant (sensitivity and intensity) [91]. In these animal models, taste responses are also assayed by recording the activity of the gustatory cells or sensory nerves, both *ex vivo* or *in vivo*, using electrophysiology and calcium imaging. These methods can also be applied to measure the activity of taste processing and reward circuits. To measure preference, choice assays are used, such as the two-bottle preference test in rodents and the flyPAD [92], FLIC [93], Expresso [94], and the CAFE/Arc [95] in insects; taste reactivity assays, which quantify facial expressions following presentation of a tastant [96] are also an hedonic measure. Food intake is assayed by keeping track of the amount of food consumed or by using feeding interactions measured by lick-o-meters as a proxy for feeding.

## Glossary

Body mass index: an approximate measure of body fat that incorporates height as well as weight
Chemosensory plasticity: changes in the chemosensory (taste and smell) cells that may impact taste sensation and preference
Chorda tympani: nerve tract (part of CN VII) that carries taste information from the tongue
Diet composition: ratio of macromolecule components in ingested food
Incentive sensitization: increased drive, or wanting, for something following successive exposures to it; usually used in the context of drug abuse
Intensity: a perceived tastant quality; higher tastant concentrations correlate with greater perceived intensities
Nucleus of the tractus solitarius: located in the hindbrain, it is the site of the first synapse for taste information from the tongue
Perception: higher-order neuronal response to a tastant, often accompanied by conscious, qualitative recognition; can be inferred in nonhuman species
Peroxisome proliferator-activated receptor γ coactivator 1α: a conserved transcriptional coactivator that regulates metabolism in response to physiological stimuli
Phospholipase C-β2: key component of a signaling cascade downstream of the TAS1R2/TAS1R3 activation; induces an increase in cytosolic calcium levels to activate TRPM5
Polycomb repressive complex 2: a conserved chromatin silencing complex that represses gene expression and writes stable epigenetic memories
Proboscis extension response: an assay that measures an insect’s innate response to stimulation of its taste organs by desirable compounds
Satiety: a physiological state lacking hunger drive; often achieved at the end of a meal
Sensation: a broad term that encompasses both the immediate sensory stimulation of the taste cells and the central processing of the taste information
Sensitivity: degree of sensation or perception in a taste system evoked by a tastant; a system with high sensitivity will respond to lower tastant concentrations
Sensory enhanced satiety: a modulation of satiety driven by sensory experience
Subesophageal zone: the region of the insect brain that receives peripheral taste inputs
Taste receptor 1 member 2: required in mammalian taste receptor cells for detection of sweet (heterodimer with TAS1R3) and umami (homodimer) compounds
Transient receptor potential channel M5: activated by PLCβ activity
